# Ability Tests Measure Personality, Personality Tests Measure Ability: Disentangling Construct and Method in Evaluating the Relationship between Personality and Ability

**DOI:** 10.3390/jintelligence6030032

**Published:** 2018-07-10

**Authors:** Patrick C. Kyllonen, Harrison Kell

**Affiliations:** Research & Development, Educational Testing Service, Princeton, NJ 08541, USA; hkell@ets.org

**Keywords:** personality, ability, constructs, methods, sources of variance, maximal vs. typical performance, high vs. low-stakes testing, objective personality tests, confidence, economic preferences, survey effort, response time, item-position effects, faking

## Abstract

Although personality and cognitive ability are separate (sets of) constructs, we argue and demonstrate in this article that their effects are difficult to tease apart, because personality affects performance on cognitive tests and cognitive ability affects item responses on personality assessments. Cognitive ability is typically measured with tests of items with correct answers; personality is typically measured with rating-scale self-reports. Oftentimes conclusions regarding the personality–ability relationship have as much to do with measurement methods as with construct similarities and differences. In this article, we review key issues that touch on the relationship between cognitive ability and personality. These include the construct-method distinction, sources of test score variance, the maximal vs. typical performance distinction, and the special role for motivation in low-stakes testing. We review a general response model for cognitive and personality tests that recognizes those sources of test score variance. We then review approaches for measuring personality through performance (objective personality tests, grit game, coding speed, economic preferences, confidence), test and survey behavior (survey effort, response time, item position effects), and real-world behavior (study time, registration latency, behavior residue, and social media). We also discuss ability effects on personality tests, indicated by age and cognitive ability effects, anchoring vignette rating errors, and instructions to ‘fake good’. We conclude with a discussion of the implications for our understanding of personality and ability differences, and suggestions for integrating the fields.

## 1. Introduction

This article reviews evidence for how cognitive ability and personality traits are integrated. There is a substantial literature that examines the correlations between measures of cognitive ability and measures of intelligence, contemporaneously [1,2], and longitudinally [3]. However, this literature almost exclusively treats scores from cognitive abilities and personality measures as pure indicators of cognitive abilities or personality traits, respectively, save for measurement error, and occasionally, as acknowledged by the inclusion of more than one measure, factorial uniqueness.

We do not review that literature.^1^ Instead, our point of departure for this article is that personality and cognitive ability are intertwined during item responses on cognitive tests and personality assessments. That is, a response to a cognitive test item typically reflects personality to some extent, and a response to a personality item typically reflects cognitive ability to an extent. The possibility that test scores reflect influences other than ability has long been recognized [7,8]. But the fact that such cross-contamination exists, to the extent that it does, complicates a number of widely held beliefs about both cognitive ability and personality, such as their relative independence, the magnitude of sub-group and country differences in personality and intelligence; the meaning of trend changes, such as maturation and early adolescent ‘storm and stress’ [9]; and the interpretation of predictive validity evidence linking personality and ability measures to educational and workforce outcomes.

The article is organized as follows. First, we review the construct-method distinction—the distinction between cognitive ability and personality constructs (or ‘traits’), and the methods used to measure those constructs. We believe these are almost always confounded, and often conflated, as indicated in, for example, the ‘personality change’ literature, which deals almost exclusively with changes in responses to a very specific kind of assessment, a self-rating Likert scale, rather than to personality per se [10,11]. We also review influences other than ability on cognitive test scores, as initially outlined by Thorndike [8], and review a general model to accommodate multiple variance sources, following a proposal by Heckman and Kautz [12]. We argue that the maximal-typical performance and high-stakes, low-stakes distinctions are critical to test score interpretation, and that motivation may be especially important in low-stakes testing. Next, we review studies that measure personality, using measures other than rating scales. These include so-called ‘objective personality tests’, and measures of choosing to put forth effort, such as the grit game and the coding speed test. We also review studies focused on economic preferences, and other studies focused on confidence measures. We review measures of construct irrelevant variance in test and survey behavior, including survey effort, response time, and item position effects. We also review measures of personality obtained in real-world behavior, such as study time, registration latency, and social media. We argue that most of these studies show that what is interpreted as a cognitive ability measure, can often be understood as measuring personality as well, and simultaneously, that it is possible to measure personality outside Likert-scale measures.

Following our review of personality determinants of performance on cognitive measures, we review instances of cognitive influences on traditional personality tests. These include age and cognitive ability effects, anchoring vignette rating errors, and ability to follow instructions to ‘fake good’ on personality tests. We conclude with a discussion of the implications for our understanding of personality and ability differences and suggestions for integrating the fields.

## 2. Construct-Method Distinction

To answer the question, “what is the relationship between personality and intelligence?” it is helpful to start with definitions. A suitable definition of personality comes from the American Psychological Association (APA), as follows: “individual differences in characteristic patterns of thinking, feeling, and behaving”.^2^ APA’s 1996 Intelligence Task Force [14] likewise provides a definition of intelligence as “<individual differences in the> ability to understand complex ideas, to adapt effectively to the environment, to learn from experience, to engage in various forms of reasoning, to overcome obstacles by taking thought”.^3^

For a combination of historical, accidental, and practical reasons, two broad approaches to measuring these two constructs have emerged and come to dominate how we think of them. For personality, the dominant methodology has to do with endorsements of descriptions of characteristic behavior, thoughts, beliefs, or attitudes. Descriptions can be trait terms or statements; endorsements can be ratings, rankings, or preference judgments between two or more descriptions; and the endorsements can be done by the self or by others—peers, teachers, or supervisors. There is a lively body literature in personality psychology on the differences between these methods, but personality is most often measured with self or peer ratings of statements using the Likert scale format [15]. The essence of the method is that it involves evaluating the target’s “characteristic patterns of thinking, feeling, and behaving” represented by descriptions.

For intelligence, the dominant method is the standardized test, with a problem and response format (multiple choice, short answer, and essay), scored as right or wrong, or in some cases, partially right. This characterizes all IQ, achievement, and selection and admissions tests such as the Armed Services Vocational Aptitude Battery (ASVAB) and the College Board’s SAT test.

So, the answer to the question of “what is the relationship between intelligence and personality?” would typically be given by a correlation between a measure of personality using the rating scale method, and a measure of intelligence based on a test. From this literature we find that those who score well on intelligence tests are slightly more likely to say that they “enjoy hearing new ideas” compared to those who score poorly, but are equally likely as their low scoring counterparts to say that they “respect others”, “pay attention to details”, “make friends easily”, or “worry about things”. Studies such as this abound in the literature [2,3,16], varying in the particular tests and in the statement endorsement method used.

However, intelligence can just as easily as personality be evaluated with the statement endorsement methodology. That is, rather than giving a test, we can ask examinees their level of agreement to statements such as “I understand complex ideas”, “I adapt effectively to the environment”, “I learn from experience”, or “I engage in various forms of reasoning to overcome obstacles by taking thought”. This is not the typical way we measure intelligence, and in fact, personality psychologists might recognize or characterize some of these statements (which were taken directly from the APA definition of intelligence, above) as measures of the ‘intellect’ facet of openness. But our point here is to distinguish the constructs (personality and intelligence) from the methods used to measure them (rating scales and tests).

Conversely, personality can be measured with tests. This is arguably more challenging than measuring intelligence with ratings, and there is not a consensus literature on how to do this. In this article, we review a number of different approaches for measuring personality without ratings.

The points here seem obvious, but we believe that there is a tendency to ignore the construct-method distinction when discussing personality and intelligence. We hear too often discussions about personality that really are discussions about examinees’ responses to Likert rating scales of personal descriptions. Similar to Boring’s [17] oft-repeated criticism of intelligence being “what the tests of intelligence test”, personality can equivalently be critiqued as “what it is that personality rating scales assess”.

## 3. Sources of Test-Score Variance

Cronbach [7], following Thorndike [8] (see also [18]), classified the sources of variance in test scores into the dimensions temporary vs. lasting, and general vs. specific individual characteristics. Lasting characteristics include personality, both lasting general (“attitudes, emotional reactions, or habits generally operating in situations like the test situation” [7], and lasting specific (“attitudes, emotional reactions, or habits related to particular test stimuli”, [7]. Temporary-general effects include health, fatigue, emotional strain, mood, and motivation, which may be referred to as state variables [19]. Temporary-specific effects include fluctuations in attention (distraction) and memory, emotions brought on by specific items, specific knowledge pertaining to an item, or item type, perhaps due to coaching and luck.

Table 1 is a modification of the Cronbach–Thorndike table, in which we add several rows (sources of variance) and we also include additional columns on how these sources of variance are treated or could be treated both in assessment design and in analysis of test scores or item responses. In general, sources of variance other than the intended construct can be labeled as construct irrelevant sources of variance and should be minimized through test design (e.g., clarifying/simplifying instructions, eliminating cues), or statistically controlled for in modeling (e.g., multitrait-multimethod, factor analysis, control variables in regression analysis).

Some of the cognitive test score variance sources are marked as personality (e.g., typical effort, anxiety) or as a state source (e.g., emotional strain and mood/emotion). We do this both to highlight the importance of personality and mood-state variance sources in cognitive testing, which is the main point of this article, but also to show that the role of these other factors has been acknowledged in psychological testing for a long time. We also provide mostly contemporary references for these factors that illustrate how they contribute to performance on tests. In this article, we examine a number of these alternative variance sources in depth.

There has been longstanding awareness of the potential for personality and mood to enter into cognitive test performance. However, the major effect of that awareness has been to be mindful of these potential sources of contamination in test design (third column), and occasionally, for particular studies to model the alternative variance sources (fourth column). For the most part, however, we argue that these potentially confounding influences are ignored.

## 4. Response Model for Cognitive and Personality Tests

Borghans, Duckworth, Heckman, and ter Weel [39] provide a general framework for measuring performance in any situation. They proposed viewing performance through the framework of the standard factor analysis model, but pointed out some of its limitations, including the arbitrary location of factors, the lack of concern for causality, the fixed nature of factors, and, particularly in personality, the problem of faking. They proposed an alternative based on predicting real world outcomes, which addresses these traditional limitations.

They also explicitly proposed that the measured traits ($Y_{l}$) in particular situations or occasions ($Y_{l}^{n}$), are only imperfect proxies for true traits ($f_{l}$), with other influences on measured traits being other related traits ($f_{\sim l}$), specific situational incentives associated with the measurement of the target trait ($R_{l}^{n}$) (e.g., high-stakes vs. low-stakes testing; rewards for performance), and the context for measuring the target trait ($W_{l}^{n}$) (e.g., contexts varying in the appropriateness for expressing the trait, situational press^4^ [41,46,47]). They argued from the model, that to measure the desired trait ($f_{l}$), it is necessary to set benchmark levels for the other influences, for example, setting common incentives, $R_{l}^{n}={\bar{R}}_{l}$, and contexts, $W_{l}^{n}=\;{\bar{W}}_{l}$, across respondents. They pointed out, as we do here, that psychologists have been negligent in setting benchmark states, with the consequence of drawing inappropriate conclusions about the generalizability of trait measures across contexts and situations. Table 1 can be seen as a list of categories and examples of traits, incentives, and contexts that influence measured performance in situations ($Y_{l}^{n}$), and the design and analysis columns represent some attempts that have been made to either set benchmark states (i.e., ${\bar{R}}_{l},\;{\bar{W}}_{l}$) or adjust for the lack of them afterwards.

From psychology, there are related frameworks for capturing the effects of testing contexts (occasions), incentives, and other influences on test scores. For example, generalizability theory [48,49] specifically identifies a universe score as an expected observed score over all observations in a universe of generalization, where the universe is defined through a set of fixed and random facets (e.g., across all raters and occasions, given some fixed incentives). Latent trait–state theory (LST) [50,51] specifically addresses the importance of latent states as well as traits on performance, by decomposing measurement error into separate latent trait and state residuals from situations and person-situation interactions. The framework also accommodates change over time.

**Figure 1:**
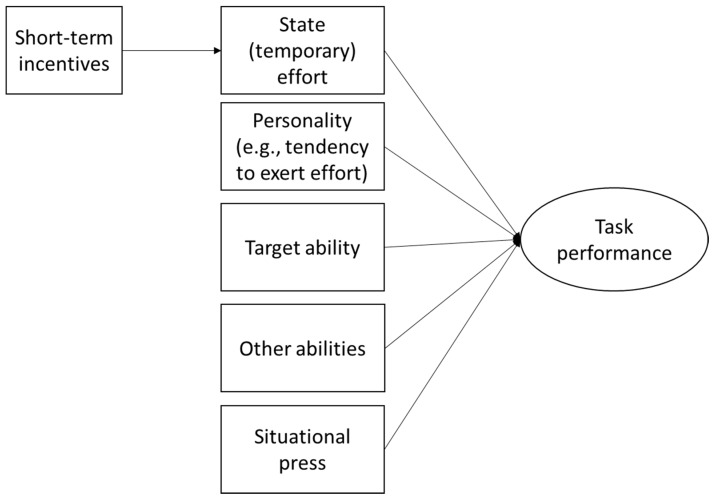
Sources of test score (task performance) variance (following Heckman and Kautz [12]).

Heckman and Kautz [12] provided a useful graphical depiction of their model, which emphasizes the point that any performance (e.g., test performance) will be a function of (a) abilities, (b) personality, and (c) motivation, and that motivation, in turn, will be a function of the incentives provided.^5^ We generalize this idea slightly to propose a range of temporary and lasting influences on task performance, including both the target ability and other abilities, state effort (influenced by short-term incentives), the general tendency to exert effort, and situational press (see Figure 1). Although just one personality trait is listed (i.e., tendency to exert effort), others could also be included (e.g., trait anxiety). This diagram can be viewed as a simplification, reflecting causal directionality, but glossing over some issues, such as multilevel relationships and interactions between factors (e.g., type of short-term incentive × personality) [53,54]. 

## 5. Maximal-Typical vs. High-Stakes Low-Stakes Distinction

Personality traits and intelligence are normally conceptualized in different ways. Personality traits are often defined in terms of typicality—stable patterns of behavior over an extended period of time [55,56,57]. If person A frequently acts in an assertive, talkative manner across a wide variety of everyday situations, she would be considered more extraverted overall than person B, who is only moderately talkative and assertive on average. However, person B, if properly motivated, may be able to act in ways more extraverted than usual, and the upper limit of person B’s extraversion may even exceed person A’s, because of situational press. Conceptualizing and measuring personality traits by their maximal expression has occasionally been considered and attempted [58,59,60,61,62,63], but the vast majority of research and theorizing treats personality traits as the average expression of a person’s behavior [64]. Consequently, personality traits are usually treated as summaries of what individuals typically do [65].

Intelligence is implicitly (and sometimes explicitly) conceptualized and measured as what people are able to do and is defined as the limit of a person’s intellectual repertoire, which can be expressed when that person is exerting maximum effort [66,67]. Cognitive ability tests are often administered under high-stakes conditions (e.g., personnel selection, university admissions), which are presumed to induce individuals to be motivated to do as well as possible on those tests and, as a consequence, demonstrate their current degree of intelligence to its fullest extent.

Just as people are capable of expressing personality traits to greater (or lesser) extents than they ordinarily do, people are also not usually motivated to express the utmost limit of their intellectual skills on an everyday basis. Consequently, there is no guarantee that individuals will demonstrate the full extent of their intelligence across the situations they encounter in their daily lives (Ackerman, 2018). For example, a person with a Ph.D. in engineering (or in English literature) will be capable of solving highly complex mathematical problems (or writing a thought-provoking essay), but may not feel motivated to do so if those problems (or essay prompts) are presented under low-stakes conditions without adequate incentives. Although the extent to which individuals demonstrate their intelligence in everyday situations has been explicitly studied in terms of dispositions [68], typical intellectual engagement [69] and through the application of ‘user-friendly’ cognitive tests [70], the majority of research and theory concerned with intelligence treats the construct as what people can maximally do intellectually [58,65,71,72].

Because intelligence is treated as the upper limit of individuals’ cognitive skills, it is amenable to being directly measured, as it is not necessary that people maintain the expression of this upper limit beyond a relatively short period of time (e.g., while taking a high-stakes standardized test). Intelligence tests can thus be conceptualized as *samples*—actual performances that directly demonstrate the construct [73,74].^6^ In contrast, because personality traits are defined as typical behavior over a long period of time, there is a view that they cannot be directly measured in conventional assessment settings. Consequently, the most frequent method of personality assessment is self-report; people are asked to complete a questionnaire about themselves, with the idea that personality traits can be indirectly measured via individuals’ self-perceptions, which are partly based on their own observations about trends in their behavior over long periods. Consequently, self-report personality surveys are actually measures of self-concept [79,80] and thus *signs*—indirect indicators of the constructs of interest [73,74]. These self-reports capture some of the shared reality of people’s actual behavior, as correlations between self-reports of personality traits and observers’ reports of personality traits range from *r* = 0.29 to *r* = 0.41 [81].^7^

The meta-analytic correlation between typical and maximal performance in the workplace has been estimated to be *r* = 0.42 [22]. Behavior on-the-job is influenced by both cognitive skills and personality (along with other constructs), and this meta-analytic correlation cannot be considered indicative of the relationship between maximal and typical behavior within either of those domains individually. Nonetheless, the relatively low correlations between behaviors in the same domain, carried out under different conditions, is intriguing and suggests the need for additional research examining the interrelations between maximal and typical expressions of intelligence and personality within and between the two domains.

## 6. Cognitive Test Performance under Low-Stakes Conditions

There is a corollary to the fact that many people do not enact the full extent of their cognitive skills on an everyday basis because they lack the incentive to do so: In testing situations where the stakes are low (e.g., laboratory experiments, nationally-sponsored learning assessments) many test-takers also lack the incentive to exert the effort necessary to perform as well as possible. Although the potential for high-stakes conditions to lead examinees to distort their responses to personality measures has been noted for decades [84] less attention has been made to the potential for low-stakes conditions to introduce construct-irrelevant variance into cognitive test scores (for exceptions see [72,85,86]). When intelligence tests are administered under high-stakes conditions, all individuals are expected to be maximally motivated and, as a consequence, cognitive ability is assumed to be the primary (and perhaps only) source of test score variance [58]. When performance on cognitive tests has little to no consequences for test-takers it is naïve to assume that all test-takers are exerting maximal effort and that subjects do not vary in whatever degree of effort they do put forth [87]: “A common assumption when studying human performance is that subjects are alert and optimally motivated. It is also assumed that the experimenter’s task at hand is by far the most important thing the subject has to do at that time. Thus, although individual differences in cognitive ability are assumed to exist, differences in motivation are ignored”.

The implications of differences in motivation for the construct validity of intelligence tests administered under low-stakes conditions have occasionally been explored over the past 70 years [88,89,90,91,92] but research in this area has intensified in the last 20 years [27,93]. Some lines of contemporary investigation have sought to demonstrate the influence of effort on test performance when the stakes are low by experimentally inducing motivation. Means of inducing effort have varied across studies but included manipulating motivational frames (e.g., “scores will be made available to employers”; [26]), offering monetary incentives [94], publicly recognizing students for their test performance [95], and providing feedback about performance [96]. Other studies have used nonexperimental procedures to study effort, such as measuring motivation via self-report [97], observational coding [98], filtering out subjects with extreme response times [99], and using person-fit statistics to detect unusual response patterns [100]. The general conclusions from these lines of research is that effort matters: Two meta-analyses have estimated a mean performance difference of 0.59 to 0.64 standard deviations between motivated and unmotivated students [98,101].

Being dispositionally motivated to achieve is related to task persistence and engagement [102], strongly related to conscientiousness [103], and even treated as an element of conscientiousness in some personality taxonomies [104,105,106]. Taken together with findings that individuals differ in their motivation to do well on tests in the absence of adequate incentives [21], this suggests that variance in scores on assessments administered under low-stakes conditions can be attributed to both intelligence and personality. When scores on such tests are judged to be “pure” indicators of cognitive skills their construct validity is compromised, as personality contributes construct-irrelevant variance [107]. However, if variance in these scores is judged to be attributable to intelligence and personality their construct validity is considerably strengthened. Indeed, test scores under low-stakes conditions can be treated as partially being measures of personality.

That a substantial portion of the variance in cognitive test scores may be attributable to personality in low-stakes settings but not high-stakes settings implies that assessment conditions (and incentives) may be an important moderator of observed associations between personality and intelligence. For instance, given that conscientious people are more likely to exert effort in general, it might be expected that the correlation between conscientiousness and intelligence test scores will be higher in non-incentivized, low-stakes conditions than in high-stakes conditions. A cursory review of studies reporting correlations between conscientiousness and ACT/SAT scores, and conscientiousness and low-stakes test scores in different samples supports this hypothesis in a preliminary way. Richardson, Abraham, and Bond’s [108] meta-analysis reports a sample-weighted correlation of −0.05 between conscientiousness and ACT/SAT, while Poropat’s [109] meta-analysis records a correlation of −0.03; the sample-weighted correlation derived from Noftle and Robins’ [110] primary study is −0.04. These values contrast with correlations reported in some studies, where intelligence tests were administered under low-stakes conditions, such as 0.29 [111] and 0.20 [112].^8^ Any attempt to understand the relationship between personality traits and intelligence must take into careful consideration the circumstances in which assessments were administered. Just as the relationship between intelligence scores and scores on a personality test administered for hiring should not be taken at face value, nor should the association between personality scores and scores on an intelligence test administered under low-stakes conditions.

## 7. Personality Measured through Performance Tests

### 7.1. Objective Personality Tests

Cattell and Warburton [116] distinguished three kinds of personality assessments, namely questionnaires (Q-data), biographical data (L-data), and tests (T-data). As noted above, almost all the research in personality has been concerned with Q-data. But in his Essentials of Psychological Testing, Cronbach [7] devoted an entire chapter to ‘Performance Tests of Personality’, tracing the history back to the Character Education Inquiry [117], which included performance tests of honesty (failing to cheat when the opportunity presented itself) and persistence (reading and marking a string of letters that formed sentences). He also reviewed cognitive style tests, in-basket tests, leaderless groups, projective tests, and other methods, which have not had a serious impact on personality testing per se (although such measures are used in applied workforce personnel selection). A problem with many of the early efforts was insufficient reliability.

A more recent treatment was provided in a special issue on Objective Personality Tests in the *European Journal of Psychological Assessment* [118]. Ortner and Proyer [119] provided a comprehensive review of objective personality tests (OPTs), distinguishing between three kinds. One is OPTs masked as achievement tests. An example is the ‘time pressure task’, in which examinees use dragging and dropping to categorize letters. The time limit gradually decreases, and the score is based on whether the examinee’s performance increases or decreases as the time limit goes down. James’ [120] conditional reasoning test (CRT) is a very different measure, but it might also be considered a measure of this type. It presents five alternative multiple-choice reading comprehension problems with two correct answers. The two correct answers reflect different world views, which are presumed to be revealed by one’s selection.

A second category is OPTs that represent real-life situations, particularly risk propensity. An example is the Balloon Analogue Risk Task (BART; [121]), in which the test taker gets more points for blowing up a balloon; the larger it gets the more points the test taker gets, until it pops, in which case all of the points are lost (test takers decide when to stop blowing up the balloon). Similar risk-taking tests have been made from decisions to cross a road [122,123].

The third category is questionnaire-type OPTs that ask for decisions. An example is one in which a problem is presented (e.g., “You are a mile away from the nearest station when the car breaks down. What would you do? If you know, make a checkmark”), which is scored for assertiveness/confidence as the latency to respond, regardless of the response.^9^

### 7.2. Grit Game

Alan, Boneva, and Ertac [20] evaluated the effects of a 10-session, after-hours educational intervention (referred to as a ‘grit intervention’), designed to promote students’ ability to set appropriate goals, and to attribute success and failure to effort rather than to factors outside their control (e.g., intelligence). They evaluated the intervention with a real effort mathematical task (a ‘grit game’), which was to find pairs of numbers that add up to 100 from a grid; they are given a target number of pairs to find (three) and a time limit (1.5 min). They could choose between a more and less difficult task (varying in the grid size), where the more difficult task paid out more (four gifts for winning vs. one gift for winning; failing to achieve the goal resulted in no gifts in either task). The intervention was successful in that it led to students seeking the more challenging version of the task, apparently to accumulate skills, which in turn led to an increase (*d* = 0.28) in performance on a standardized test. This study illustrates a couple of principles. One is that the relationship between personality (in this case, grit, or the tendency to select challenging goals and exert effort) and intelligence (performance on the math test) is not fixed, but it can be modified by an intervention focusing on beliefs about the importance of effort. Second, it is possible to measure a personality construct by way of a decision behavior related to a game-like task.

### 7.3. Coding Speed Test as a Measure of Personality

Segal [21] argued that what a test measures depends on stakes; high-stakes tests measure cognitive skills, but low-stakes (i.e., unincentived) tests can measure both personality (intrinsic motivation and the tendency to exert effort) and cognitive skills. A particularly good indicator of personality for a test would be one in which the knowledge requirements are minimal, reducing the confounding effects of knowledge on performance. She argued that the Armed Service Vocational Aptitude Battery’s (ASVAB) coding speed test, which requires examinees to match common words with four-digit numbers by scanning a test form, satisfies the low knowledge requirement. The coding speed test (along with other ASVAB tests) was administered in the National Longitudinal Study of Youth (NLSY), with no incentives (therefore, low stakes) and it was found that scores on the test were correlated with earnings 23 years later, whether or not controlling (through regression) for the non-speeded portion of the ASVAB (which would measure cognitive ability), and also controlling for educational attainment, suggesting that it is personality and not cognitive ability component of coding speed that relates to workforce success. To buttress this claim, Segal also showed that (a) recruits, who take coding speed under incentivized conditions (military entrance), scored higher than NLSY participants, despite having less education; (b) in an experiment, about a third of the participants responded to incentives by increasing their performance (call this the unmotivated group, in that they need incentives to respond well), the others did not (call this the intrinsically motivated group, since they perform well regardless); (c) both groups (unmotivated and intrinsically motivated) had equal SAT scores; (d) there were more females in the intrinsically motivated group; and (e) males in the intrinsically motivated group had higher conscientiousness scores than the remaining males.

### 7.4. Economic Preference Games

Almlund, Duckworth, Heckman, and Kautz ([125], Table 6) proposed a set of tasks from behavioral economics research measuring time (delay discounting), risk (aversion), and social (leisure, altruism, trust, reciprocity) preferences. Such preferences are assumed to be fairly general, and lasting, therefore by our definition, can be thought of as personality factors. These have tended to correlate, but only weakly, with survey measures of the Big 5^10^. Big 5 items that would appear to reflect preferences include risk tolerance (e.g., “I take risks”, “I avoid dangerous situations”), typically considered a facet of extraversion; time preferences (e.g., “I put off unpleasant tasks”, “I avoid responsibilities”, “I get chores done right away”) typically considered a facet of conscientiousness; and social preferences (e.g., “I love to help others” and “I trust others”) typically considered a facet of agreeableness.

In a series of studies, Falk and colleagues [127,128] developed a battery of tasks designed for surveys for measuring risk, time, and social preferences—specifically, risk aversion, future time discounting, trust, altruism, and positive and negative reciprocity—which they called the preference survey module. They implemented this module as part of the Gallup World Poll, which was administered to 80,000 individuals in 76 countries.

The module was developed as follows. First, an experimental measure for each of the six preferences was administered; these measures involved real money and payouts. For example, a risk-taking measure asked whether a respondent preferred a lottery or a safe option, with varying amounts of money. A time preference measure asked whether a respondent preferred an immediate or delayed payout with varying amounts of money. A trust measure asked how much money a respondent would give to another in an investment game (i.e., anticipating that the other would return some of that money). A negative reciprocity measure determined what the minimal amount of money a respondent would accept from another would be, before rejecting the offer (in which case both receive no money, i.e., an ultimatum game). 

Next, numerous survey items were administered to the same respondents, and regression analyses were used to select the two survey items that best predicted performance on the experimental measures. The survey items were of two types, qualitative and quantitative. Qualitative items were typical Likert style personality items, such as “Are you a person who is generally willing to take risks, or do you try to avoid taking risks?” (risk-taking), “How willing are you to give up something that is beneficial for you today in order to benefit more from that in the future?” (time preference), “As long as I am not convinced otherwise, I assume that people only have the best intentions” (trust), and “Are you a person who is generally willing to punish unfair behavior even if this is costly?” (negative reciprocity). The quantitative measures were typically survey versions of the experimental measure, which asked respondents what they would do in a situation. For example, a respondent would be given a set of choices of payments, they would prefer “today” vs. “in 12 months” (e.g., 100 dollars today vs. 120 dollars in 12 months) (time preference). Or they would be told “Suppose you won a lottery for $1000, how much would you give to charity?” (altruism).

Falk et al. [128] estimated the correlations between performance on the experimental tasks, and a best composite of the two survey items, for each dimension. The correlations ranged from 0.38 (negative reciprocity) to 0.58 (time preference), which are reasonably high given that each was measured by only two survey items, and the test–retest correlations between the game tasks ranged from 0.35 to 0.67.^11^ Administering the two-survey-item-per-dimension module as the Global Preference Survey (within the Gallup World Poll), resulted in many interpretable findings, such as women being more risk averse than men, with stronger social dispositions; risk taking being lower with age; cognitive skills correlating with time preference and risk taking; time preference (future time orientation) being related to educational attainment and savings; risk-taking being associated with smoking and self-employment; and social preferences being related to donating, volunteering, and helping others.

### 7.5. Confidence

In a series of studies, Stankov and colleagues [129,130,131,132] have argued that confidence is one of the most powerful non-cognitive predictors of academic achievement, as well as other outcomes. In their approach, confidence is measured following the completion of an item response, for example, after a vocabulary, mathematics, and cognitive reflection test [133], or a progressive matrices item. Then, a respondent is presented with a confidence scale (“How confident are you that your answer is correct? Choose one—0%, 10%, 20%, 30%, 40%, 50%, 60%, 70%, 80%, 90%, or 100%”). Up to half the variance in performance is captured by this measure [130]. Several cycles of the Program for International Student Assessment (PISA) have also used a confidence measure (called ‘mathematics self-efficacy’) and similarly, it is among the highest correlates of achievement (average within country correlation, *r* = 0.43; [134]. There is evidence that confidence, measured this way, is somewhat independent of the ability measured by the particular test item [135], which supports the hypothesis that there is a general, lasting trait of self-confidence, which can be measured in the context of specific cognitive ability test items. Although no study, to our knowledge, has directly compared a survey approach to measuring confidence (e.g., a Big 5 facet of extroversion), the achievement correlations given by the experimental measure are higher than meta-analytic estimates of achievement correlations given by survey measures [109].

## 8. Personality Measured through Test and Survey Behavior

### 8.1. Survey Effort

The effort one puts forward in responding to a survey, both by returning it, and by completing all items on that survey, might be understood as an indicator of either a temporary or lasting characteristic of that individual [136,137,138]. Item response rates were examined by Hitt, Trivitt, and Cheng [139], who examined six large-scale longitudinal surveys of adolescents. They found significant relationships between item response rates and educational attainment (a one standard deviation [SD] increase in item response rate was associated with 0.11 to 0.33 additional years of education); this relationship held as significant in four of the six datasets, even after controlling for a number of other factors, including cognitive ability. The fact that cognitive ability, as measured by tests, attenuated the relationship could be seen as evidence that test scores already include variance associated with effort.

A question is whether lack of effort, indicated by skipping background questions, is related to conscientiousness or other Big 5 personality factors, measured by surveys. Zamarro, Cheng, Shakeel, and Hitt [140] examined behavior within the Understanding America Study (UAS), a 30 min panel survey of 6000 households in which respondents are paid $20 per survey. Specifically, they examined item nonresponse rates and careless answering. Careless answering was defined as a kind of person misfit statistic, in which each item response on a scale was regressed on the average score from the remaining items on a scale, and a standardized residual was taken to represent misfit. Misfit was averaged across several scales to form a composite careless answering variable. They found significant but low correlations between nonresponse rates and careless answering with Big 5 variables. More importantly, they found that careless answering and item nonresponse independently were associated with educational attainment, more so than Big 5 personality traits measured by a survey. In addition, careless answering was associated with labor market outcomes (earnings), although item nonresponse was not.

Besides careless answering, returning the survey itself may be taken as an indication of effort. Cheng, Zamarro, and Orriens [141] examined return rates on the same Understanding America Study (UAS). They found that those returning surveys were more conscientious and less open, after controlling for a wide variety of demographic characteristics,^12^ supporting the typical-effort interpretation of the conscientiousness factor.

### 8.2. Item Position Effects

It is commonly assumed that an item’s difficulty (e.g., the percentage of test takers who get the item right) is not affected by whether that item is administered early or late in a test (this assumption is implicit in the expression “item difficulty”, which is not conditioned on item position). That assumption is sometimes relaxed, in acknowledgement of warm-up and fatigue effects, which may increase item difficulty [142]. A common remedy is creating two or more test forms that vary item position, thereby averaging out the item position effects. Nevertheless, the existence of item position effects is a reflection of noncognitive influence (warming up, being mentally fatigued) on a cognitive test score.

One question concerns the severity of item position effects. This seems to vary depending on circumstances, but there certainly is considerable evidence for them. Albano [143] found that items in the middle of a first-grade reading achievement test were 8% more difficult than they were at the beginning of the test (*N* = 93,000+). He found similar effects for the Graduate Record Exam (GRE) (*N* = 5000+). In both studies there was item heterogeneity in the sense that items varied in their susceptibility to position effects; on the GRE the range was from a proportion correct decreasing by 0.17 to increasing by 0.03 in early vs. late item positions. 

Another question is whether there are group or individual differences in susceptibility to the item position effects. Debeer, Buchholz, Hartig, and Janssen [144] (see also [31,145]) examined data from the low-stakes Program for International Student Assessment (PISA) 2009 reading assessment (*N* = 460,000+, 65 countries), using an item-response theory (IRT) model that included item position effects, which enabled modeling test-taker effort (i.e., less susceptibility to item position is assumed to indicate more [consistent] effort). They found both a general decrease in effort across countries, and large individual, school-level, and country-level differences in the decrease of examinee effort over the course of the test. The amount of decrease was associated with the overall performance level. For example, students from Finland, which is a high (average) performing country, showed a relatively small decrease in effort (*d* = −0.09), whereas students from Greece, which is a lower performing country, on average, showed a larger decrease in effort over the course of the test (*d* = −0.28). There were also school effects on persistence, supporting a positive relationship between persistence and ability (schools with higher ability students also are ones with higher-average-persistence students). The large lesson here is that differences in PISA scores between countries at least partly reflect noncognitive (persistence) differences between students in those countries. This result was replicated at the student level in a large-scale German achievement study [146], using a survey approach to measure effort as a predictor of change in item difficulty during the test.

### 8.3. Response Time

Another indicator of effort on a cognitive ability test, particularly one given under low-stakes conditions, is response time. The idea is that if examinees are responding quickly, for example, in less time than it takes to read the question, they are not putting forward adequate effort for solving the problem. Wise and Kong [99] proposed a measure of response-time-effort (RTE) indicated by the proportion of items for which the examinee takes adequate time to respond, that is, more time to respond than a low threshold (which can be determined in various ways [147], but typically below a second or two, depending on the task). This measure has been shown to relate to test performance as well as other outcomes [148]. Students who display less response-time-effort tend to do more poorly on the assessment. Lee and Jia [149] developed a response-time-effort method to investigate student effort on the National Assessment of Educational Progress (NAEP).

Wise and Gao [150] proposed a broader measure of test taking effort on computer tests, which they refer to as response behavior effort (RBE). In addition to rapid guesses (the response measured with RTE), they proposed rapid omits and rapid perfunctory answers on constructed response items. In all of these cases, they used a threshold of 10% of the average time test takers spent on the item, or 10 s (whatever was lower) as the threshold to defined rapid guessing, omitting, or perfunctory answering. They applied this method to the Office of Economic Cooperation and Development’s (OECD) 2013 PISA-Based Test for Schools (PBSTS), and found that about 5% of all of the items showed a RBE value of less than 0.90, due to rapid guesses (71% of non-effortful responses), rapid omits (19%), and rapid perfunctory answers (10%). The highest achievers (by quartile) displayed the highest behavior effort (75,157 solution behavior responses/75,216 responses total = 99.9%; vs. the lowest quartile at 95.3%, computed from information in their [150] Table 3). However, it was also true that for those items in which the test taker did display solution behavior, there still was a difference of 27% vs. 72% correct for the lowest vs. highest quartile.

## 9. Personality Measured through Real World Behavior

### 9.1. Study Time

How students choose to spend their out-of-school time can be thought of as a noncognitive factor that is integral with achievement, as indicated by test scores. Time spent on homework for example, is likely to boost achievement [151]. McMullen [152] estimated that one additional hour of homework per week translated to an improvement in mathematical achievement by 0.24 standard deviations, and that this was even higher for low performing students and schools. Being randomly assigned into a homework required (vs. not-required) group was found to boost test scores, grades, and retention [153]. However, the issue here is whether choosing to do homework could be seen as a personality factor (tendency to put forth effort). A study using time-diary data found that an extra hour of homework per night increased the probability of attending college by 5 percentage points (for males) [154]. However, the authors suggested that this homework effect may be due to an omitted variable (e.g., motivation), based on an instrumental variable analysis in which day of the week (e.g., surveyed on a Friday vs. not) and season (e.g., football season or not) were treated as instruments. 

### 9.2. Registration Latency

Richardson, Abraham, and Bond [108] identified procrastination, measured by survey items (e.g., “I generally delay before starting on work I have to do”), as among the highest noncognitive correlates of college success (rho = −0.25), as defined by the grade-point average. Novarese and di Giovanni [155] examined a performance measure of procrastination, registration latency for college (law school in Italy), which is defined as the time between when a student was first eligible to enroll and when the student actually did enroll (before the deadline, near the deadline, or after the deadline, which involved paying a late fee). They found that late registering students were more likely not to complete the first year, less likely to graduate, had poorer performance, passed fewer exams, and received fewer credits. They found that this same pattern held for late registration in years two and three. A question is whether this kind of procrastination is a temporary, one-off characteristic, perhaps due to circumstances, or a more lasting one. They found some evidence pointing to it being a lasting characteristic; the correlation between procrastination from year to year (for the first five years, ignoring the first year, which was a bit of an outlier), ranged from 0.33 to 0.47, suggesting a relatively stable indicator. Interestingly, promptness averaged over years two and three, which was found to be correlated to a performance measures (number of exams passed, *r* = 0.42), was only modestly related to a self-report prompt, “I procrastinate” with a 0–10 response scale (*r* = −0.22), which itself had a lower correlation with the number of exams passed.

### 9.3. Word Use, Office Appearance, and Facebook Likes as Personality Measures

That personality does not have to be measured by Likert scale self-reports has been explored in various studies. Fast and Funder [156] examined the words used in one hour life history interviews, and found a number of moderately high, interpretable correlations between particular kinds of words used (e.g., certainty words, such as ‘absolutely’), and responses on a personality measure (e.g., “is facially and/or gesturally expressive”, “is verbally fluent”). Gosling, Ko, Mannarelli, and Morris [157] had observers view people’s offices and workspaces (when the occupants were not there), and then completed personality ratings of the offices’ occupants. A separate group of coders coded 43 offices’ features, such as their neatness. A number of suggestive correlations were found, such as conscientiousness being related to neatness, openness related to distinctiveness and unconventionalness, and so on. A similar analysis, with similar findings, was conducted on bedrooms [156]. 

Kosinski, Stillwell, and Graepel [158] explored the relationship between Facebook likes and various characteristics of Facebook users. They reduced a binary matrix of 55,000 Facebook likes (1 = the user indicated a ‘like’ for photos, friends’ status updates, sports, books, web sites) to 100 components using singular value decomposition, then used the resulting components to predict a variety of user characteristics, such as age, gender, personality, intelligence, relationship status, political views, and religion, using linear or logistic regression analysis (they obtained personality and intelligence through a special app). They found that for the continuous variables, age (*r* = 0.75), size of Facebook friendship network (*r* = 0.47), openness (*r* = 0.43), extraversion (*r* = 0.40), and intelligence (*r* = 0.39), were fairly well predicted; for the categorical variables, gender (AUC = 0.93), sexual orientation (AUC = 0.88, 0.75), political party (AUC = 0.85), and race (AUC = 0.96) were well predicted, with other outcomes (drug, alcohol, cigarette use, relationship status), moderately predicted. 

Youyou, Kosinski, and Stillwell [159] followed up this study with the administration of a longer personality (self-ratings) survey to 86,000 Facebook users who completed the survey on themselves and on several peers. They found that Facebook likes correlated more highly with personality self-reports (*r* = 0.56) than others’ ratings did (*r* = 0.49). Further, they found that Facebook likes correlated more highly with a variety of life outcomes (e.g., substance use, political attitudes, health) than self-ratings did.

## 10. Ability Effects on Personality Measures

To this point, we have focused on the influences of personality state and trait variables on cognitive test score performance, to make the point that cognitive test scores cannot be unambiguously attributed to cognitive skills. Instead, they represent a mixture of cognitive and noncognitive influences. In this section, we address the opposite question, “To what extent do cognitive factors influence responses to personality assessments?”

### 10.1. Age Effects

There is an established literature on personality change, suggesting that personality generally gets better with age. That is, based on rating scale responses, from young to later adulthood (ages 21 to 60), conscientiousness and agreeableness increase over time and neuroticism declines [160]. However, during the earlier years, from age 10 to 20, as young adults grow in sophistication in understanding language and human nature, the picture is more complex. Responses to rating scale items become more reliable from 12 to 18 years old [161], the Big 5 factor structure becomes more differentiated and closer to the adult structure over that period^13^ [162], and below age 13 (from age 10), the alignment of items to their appropriate factors (with respect to the adult structure) deteriorates substantially. In general, coherence (mean inter-item correlation of items measuring a factor) increases substantially from age 10 to 20, and differentiation (mean inter-scale correlations, controlling for unreliability) goes down. This pattern is exactly what would be expected if cognitive ability played a role in responding to personality rating-scale items. In this way, personality scores partly reflect cognitive ability differences.

### 10.2. Cognitive Ability Effects

The differentiation hypothesis of cognitive ability is based on the idea that the role of the general factor diminishes during development (or with increases in ability), dominating early childhood (or, at low ability levels), but becoming increasingly less important with development (or at high ability levels), as the role of specialized abilities (e.g., verbal vs. spatial) become relatively more important. The support for this hypothesis is mixed [163], but a question here is whether there is a similar differentiation in personality. An interpretation of age or ability-related personality differentiation is that a lack of differentiation could be due to a person’s lack of cognitive ability to comprehend personality descriptions and properly differentiate levels of agreement with those descriptions.

Mõttus, Allik, and Pullman [164] found that out of 35 personality scales (Big 5 plus facets), reliability was significantly higher for high ability groups than for low ability groups for seven of those scales, and nominally higher for 30 of the 35 scales^14^. Correlations between scales were also higher for the low ability group than for the high ability group. Of the 10 Big 5 intercorrelations, 8 were nominally higher in the low ability group; this was also seen in the size of the first principal components (22% vs. 27% of the variance for high vs. low ability groups, respectively). Based on findings from a similar study, Allik, Laidra, Realo, and Pullman [165] concluded that some younger children lacked the “developed abilities required for observing one’s own personality dispositions and for giving reliable self-reports on the basis of these observations”. It is certainly possible and desirable to reduce the complexity of items in order to reduce the effects of cognitive ability [166], but our point here is simply that, in general, cognitive ability plays a role in responding to personality items.

In another manifestation of the importance of ability in responding to personality surveys, adults are quite capable of ‘faking’ responses to personality scales (particularly, Likert type scales) to present a favorable impression. The degree to which they are able to do so is related to cognitive ability [167]. 

### 10.3. Faking on Personality Tests

The typical rating scale format of a personality test enables ‘faking’. That is, to convey a positive image of oneself, it is possible to “strongly agree” with positive statements (e.g., “I work hard”) and “strongly disagree” with negative ones (e.g., “I am lazy”), regardless of one’s personality. In fact, there is mixed evidence on the extent to which respondents actually do this, some suggesting respondents do not often fake [168], others suggesting they do [169,170].^15^ But for our purposes here, we focus on a category of studies in which respondents are asked to “fake good”. Being able to fake good indicates a sophistication about how responses are interpreted by potential decision-makers (e.g., hiring authorities, admissions committees). If there is differential ability to fake good, and that ability is related to cognitive ability, then that suggests that cognitive ability can have a direct influence on the responses to personality tests. A meta-analysis of studies that instructed respondents to fake good (or bad) suggests that indeed respondents are quite capable of doing so [172]. Effect size estimates for the Big 5 factors ranged from 0.48 (with Agreeableness) to 0.65 (with Openness), in between-subjects designs, slightly larger in within-subjects designs (instructions to fake bad resulted in effect sizes two to three times greater, in the other direction). However, respondents high in cognitive ability are particularly able to fake good, by roughly one half of a standard deviation, compared to low cognitive ability respondents [173]. This suggest that personality tests at least partially measure cognitive ability, depending on conditions, particularly incentives, and according to Griffith and Converse [170], personality tests do so.

### 10.4. Anchoring Vignettes as a Window into Psychological Understanding

The anchoring vignettes technique is a method for increasing comparability between respondents on rating scale measures, by having respondents rate both themselves and others, described in vignettes, on the same items [174]. Anchoring vignettes were included in PISA 2012 to address the problem of response style differences between countries in international comparisons [175]. The key for including anchoring vignettes as part of the discussion on the role of cognitive ability in personality testing is related to the task of rating vignettes. In PISA 2012, two sets of vignettes were included, one concerned with the dimension of classroom management, one with teacher support. Each had vignettes designed to be either low, medium, or high on the targeted dimension. For example, the high, medium, and low teacher support vignettes were, (a) “Ms. <a> sets mathematics homework every other day. She always gets the answers back to students before examinations”; (b) “Mr. <b> sets mathematics homework once a week. He always gets the answers back to students before examinations”; and (c) “Ms. <c> sets mathematics homework once a week. She never gets the answers back to students before examinations”, respectively. Following each vignette, students were asked how much they agree with the statement, “Mr./Ms. <x> is concerned about his/her students’ learning”. They could answer “strongly agree”, “agree”, “disagree”, or “strongly disagree”? Typically, students’ responses to the vignettes align with the vignettes intended trait location, so that they are more likely to agree that the high vignette teacher is “concerned about students’ learning” than the low vignette teacher is. But student responses are not always aligned with the intended vignette location. In fact, cognitive ability (whether measured by mathematics, reading, or problem solving) was (negatively) associated with either assigning two vignettes the same rating (‘ties’) or rating the intended higher vignette lower than the intended lower vignettes (‘misorderings’), with effects sizes ranging from about a half a standard deviation to about 0.8 standard deviations (see [176], Tables 10 and 11, pp. 29–30; k = 52 countries, N ~ 250,000).

## 11. Discussion

This article is a contribution to a special issue of the *Journal of Intelligence* on the integration of personality and intelligence, which invited contributions to “bring these two traditions (personality and intelligence) back to the discussion table and to underscore the relevance of an integrative perspective for both individual differences and developmental research” [177]. A central theme of this article is that, wittingly or unwittingly, intelligence researchers are already studying personality, and personality researchers are studying intelligence. 

Another theme is that, while construct and method are typically confounded—intelligence is measured with tests and personality is measured with Likert scale self-reports—in principle, they are separable, and failing to acknowledge the construct-method distinction results in dubious conclusions, such as the highest personality correlate of intelligence is the openness/intellect factor. That conclusion could nearly as justifiably be restated as the method effect for measuring intelligence is observed directly as the correlation between the openness/intellect factor (i.e., self-reports of one’s intelligence) and intelligence test scores.

We have several suggestions for how personality and intelligence research and researchers can move forward together. First, we believe that the constructs of intelligence and personality are viable. Carroll’s [66] cognitive ability taxonomy, the Big 5 framework [160], and economic preferences [125], should be thought of as useful delineations of skills that people develop with schooling and experience, and apply when making decisions and acting on them. The view of personality as a skill strikes some psychologists as odd, but it has been embraced by policy makers. Consider the title of an op-ed in the *New York Times* by a prominent U.S. Senator, “We need immigrants with skills. But working hard is a skill” [178]. The mistake is to conflate abilities, personality factors, and preferences with the methods used to measure them. Researchers should acknowledge, or attempt to control for ancillary sources of variance in these measures.

Second, as psychology and measurement psychology in particular has been recognized for a long time, it is useful to include multiple measures of a construct to unconfound the construct from its measurement. For new surveys, this might include supplementing Likert scale measures with additional behavioral, or ‘objective’ measures. And fortunately, as the literature review above shows, there may already exist multiple measures of constructs in extant survey datasets. For example, large-scale achievement surveys, such as NAEP and PISA, have been and can continue to be analyzed for indications of personality traits, such as tendency to put forth effort, by analyzing achievement item response times, item position effects, and the like. There are a large number of potential datasets that could be mined for personality indicators other than Likert scale measures.

Finally, we suggest expanding the editors’ call to bring personality and intelligence researchers back to the discussion table to include behavioral, labor, and education economists. As we hope this review demonstrates, economists have already made significant contributions to our understanding of the integration of personality and intelligence. There are the beginnings of integration with occasional special conferences [179], National Academies reports [180], and papers published in economics journals and handbooks [125]. Collaborations published in psychology journals, such as the *Journal of Intelligence* would be useful moving forward.

## Figures and Tables

**Table 1:** Sources of cognitive test score variance, including personality trait and state variance.

Sources of Cognitive Test-Score Variance	Examples	Design Treatment	Analysis Treatment
**I. Lasting, general characteristics (lasting person characteristics that pertain to performance on this test and tests like it)**			
1. Target construct of the test	general cognitive ability, verbal ability	lengthen test to extent feasible	true score variance
2. Other cognitive factors that might influence test scores	reading, vocabulary, related cognitive factors	minimize role of other factors	factor analysis; MTMM
3. General test-taking skills	comprehend instructions, test-wiseness	test coaching, practice tests	typically ignored
4. Skill with the test’s item types	multiple-choice vs. short-answer	vary item type	ignore or model
5. Personality—tendency to typically exert effort	grit game ^1^; picture-number ^2^, maximal-typical performance distinction ^3^	-	predict important outcomes
6. Personality—lack of anxiety	(lack of) test anxiety ^4^	anxiety training	ignored
7. Personality—managing time in a time-limit test	running out of time on a standardized test ^5^	provide clocks, warnings	time-accuracy models
**II. Lasting, specific characteristics (lasting characteristics that pertain only to this test or item subset)**		-	-
1. Skills required by particular item types	mode (PBT, DBT), response format (MC, CR)	multiple methods	ignore or model
2. Skills required by the particular content sample	form differences	create parallel forms	measurement error
3. Personality—Effort inducing states due to test conditions	computer-based assessments, incentives ^6^	make tests/items engaging	ignored or researched
4. Personality—Emotional state induced by test stimuli	math anxiety ^7^, stereotype threat ^8^	minimize inducements	ignored or researched
**III. Temporary, general characteristics of the individual (pertain to the whole test and tests like it, but only for a short while)**			
1. Temporary health, fatigue, emotional strain	poor performance due to being ill, sad, tired	allow retest	discard all but highest score
2. Environment effects	poor performance due to noisy/hot room	allow retest, venue flexibility	discard all but highest score
3. Level of practice on skills required by tests of this type	novel test format/content	provide pretest practice	model growth/dynamic testing
4. Personality—Effort-inducing states	motivation incentives (feedback, payments) ^9^	provide incentives to all	typically ignored
5. Personality—Emotional states	stressors (high stakes, fear of failure) ^10^	anxiety training	typically ignored
**IV. Temporary, specific characteristics of the individual (pertain only to this test or item subset, and only this time)**			
1. Personality—Changes in fatigue/motivation over the course of a test	Item position effects ^11^	minimize test length, make test more engaging	error or model
2. Personality—Emotional reaction to item response/feedback	discouragement/slowdown after item failure ^12^, “entity” theory of intelligence ^13^	Content, sensitivity, fairness reviews	error
3. Fluctuations in attention and memory	mind wandering ^14^	make test/items more engaging	error
4. Unique skill or knowledge of these particular items	effects of special coaching ^15^, prior exposure ^16^	test coaching, practice tests	error or part of construct
5. Mood/emotion State induced by item(s)	test item invokes a negative emotion ^17^	content, fairness reviews	error or part of construct
6. Luck in the selection of answers by guessing	guess correct answer	avoid MC or provide many options	error, guessing correction

NOTES: Table adapted from Thorndike ([8], p. 73), Cronbach ([7], p. 175), Stanley ([18], p. 364). MTMM—multitrait multimethod model; PBT—paper-based test; DBT—digital-based test; MC—multiple-choice test; CR—constructed response (short answer) test; SEE—standard error of equating; RT—response time. ^1^ Alan, Boneva, and Ertac [20]; ^2^ Segal [21]; ^3^ Beus and Whitman [22]; Sackett, Zedeck, and Fogli [23]; ^4^ Hembree [24]; ^5^ van der Linden [25]; ^6^ Liu, Bridgeman, and Adler [26], Finn [27]; ^7^ Hembree [28]; ^8^ Steele and Aronson [29]; ^9^ Liu, et al. [26], Finn [27]; ^10^ Beilock [30]; ^11^ DeBeer and Janssen [31]; ^12^ Rabbit [32]; ^13^ Mueller and Dweck [33]; ^14^ Kane and McVay [34]; Terhune, Croucher, Marcusson-Clavertz, and Macdonald [35]; ^15^ Powers and Rock [36]; ^16^ Irvine [37]; ^17^ Eich [38].

## Footnotes

1 But see [4,5,6] for recent reviews.

2 See also Roberts [13], who defines personality as “relatively enduring patterns of thoughts, feelings, and behaviors that reflect the tendency to respond in certain ways under certain circumstances”.

3 It is worth noting that such definitions often include references to “individual differences”. Individual differences should be thought of as a method for identifying the factors discussed here, but it is not the only method. Training effects (of intelligence or personality) are non-individual-differences methods, as are artificial intelligence approaches (e.g., building expert systems).

4 Situational press refers to the reduction in trait variance due to situational constraints [40]. H. A. Murray [41] distinguished alpha (consensual, objective) and beta (subjective) press. Related concepts include situational strength [42], personality-situation congruence [43], situational construal [44], and moderated-mediation models [45], with situations as a moderator, and personality as a mediator.

5 An anonymous reviewer pointed out that motivation itself may be multidimensional [52].

6 Although this often how high-stakes intelligence tests are conceptualized, some have argued that they are indirect, as the scores are only the results of unobserved cognitive processes [75,76,77,78].

7 Self-reports of intelligence are also somewhat accurate, as a meta-analytic estimate of the correlation between self-estimates of intelligence and intelligence test scores was *r* = 0.33 [82]. Similarly, self-reports of typical intellectual engagement and scores on intelligence measures have been found to correlate from *r* = 0.43 to *r* = 0.50 [83].

8 Other studies report much smaller relations between conscientiousness and scores on cognitive tests given under non-incentivized conditions [83,113]. Further complicating matters, a meta-analysis showed a correlation of 0.14 between achievement motivation and ACT/SAT [114]. Moreover, using ACT and SAT as the “gold standard” for high-stakes cognitive ability tests likely introduces complications due to range restriction and selection bias [115].

9 Another type of “objective measure” that could be included here are personality measures based on the behaviors individuals who are high or low on a trait report doing. An example is the Behavioral Indicators of Conscientiousness (BIC) measure [124]. However, these items end up being almost indistinguishable from typical Likert-rating personality items.

10 The Big 5 is a prominent dimensional analysis of personality, positing that five orthogonal factors—Extraversion, Agreeableness, Conscientiousness, Neuroticism, and Openness—account for item responses on personality surveys and predict real-world outcomes [126].

11 Using the standard correction for disattenuation, *r_x_*_’*y*’_ = *r_xy_*/(*r_xx’_r_yy_*)^1/2^, and numbers supplied in Falk et al. [128], we estimate the partly disattenuated correlation to be 0.53, 0.65, 0.77, 0.53, 0.71, 0.46, for risking taking, time, trust, altruism, positive and negative reciprocity, respectively, assuming perfect reliability of the composite.

12 Survey returners also were higher cognitive ability, more likely female, native born, not employed, and African American, controlling for many background factors.

13 This is true regardless of whether acquiescence is controlled for or not; acquiescence (“Yea-saying”) can be controlled by within-person standardization, or ipsatizing.

14 The authors argued that although there were differences as we note here, they did not support the conclusion that “personality differs substantially across ability groups” ([163], p. 155). However, our argument is simply that cognitive ability difference contributed to differences in personality responses, which is what the authors found.

15 Most of this literature is based on a rating-scale response format. Forced-choice methods appear to limit faking susceptibility [171].
